# The value of tourism public opinion management in social governance: A study on the impact of electronic word-of-mouth perception on people’s livelihood well-being

**DOI:** 10.3389/fpsyg.2022.1081960

**Published:** 2022-12-20

**Authors:** Xiuxiang Li, Yingqi Wu, Yi Jiang

**Affiliations:** School of International Economics and Trade, Jiangxi University of Finance and Economics, Nanchang, China

**Keywords:** social governance, tourism public opinion, electronic word-of-mouth perception, livelihood well-being, individual characteristics, environmental elements

## Abstract

In the process of tourism market upgrading and tourism iteration, tourism companies will face a more complex public opinion environment. Designing a socialized public opinion management method for tourism with social governance to improve people’s livelihood and well-being has become the primary concern of the tourism industry. Therefore, the existing literature has extensively focused on the role and influence of public opinion word of mouth from the perspective of tourist or consumer behavior. However, moderating role of individual tourist characteristics and environmental elements has not yet been deeply explored. Therefore, integrating with the background of the social media, this study examines how electronic word-of-mouth (eWOM) is related to the well-being of people’s livelihood, and explores the interaction between individual characteristics and eWOM perception in Study 1. Furthermore, in Study 2, we provided novel boundary conditions, namely environmental elements (i.e., physical, social and historical environment). We used mixed methods (i.e., quantitative and experimental designs) to reveal that tourists’ perceptions of eWOM have a significant positive effect on tourists’ well-being experience. The results show that tourists’ eWOM perception has a significant positive impact on their well-being experience. In addition, individual characteristics and environmental elements showed significant moderating effects between eWOM and well-being of people’s livelihood. This study discusses the theoretical and practical implications, exploring the value of tourism public opinion management in social governance centered on tourists’ eWOM perception, which helps tourism companies to effectively prevent and resolve risks affecting social harmony and stability in the field of cultural tourism and create a safe and stable cultural tourism market environment.

## Introduction

In recent years, with the continuous socio-economic development and the improvement of people’s living standards, tourism has a promising future (Li and Katsumata, 2020; Mamirkulova et al., 2020) Tourism has increasingly become an important starting point for enhancing social civilization and national cultural soft power (Wu et al., 2021). Economically, tourism is one of the largest sources of export earnings and an important provider of job markets in many countries (Çakmak and Çenesiz, 2020). For example, in particular, some developing countries are using it as a means of economic development to carry out various economic development activities (Cannonier and Burke, 2019). The rise of social media has revolutionized the travel industry. People post their feedback on the places they visit on the Internet, while others seek such information on the Internet. This has multiplied the interest and value of electronic word-of-mouth (eWOM). Additionally, the intent to use social networking sites in future travel-related decisions was the most prominent factor (Tran et al., 2019). However, in the process of Internet development and tourist iteration, tourism enterprises are faced with a more complex public opinion environment (Bodolica et al., 2021). For tourist attractions and tourism enterprises, controlling the excessive emotionality of online public opinion is the difficulty of tourism public opinion management (Mao et al., 2021). It is precisely because negative public opinion not only squeezes the space for true opinion, but also affects the ability of tourists to discriminate, providing a breeding ground for false rumors (Williams et al., 2022), adversely affect the further development of tourism (Mkono et al., 2020). Therefore, the tourism industry needs a social approach to manage the public opinion, such as tourists’ eWOM, and individual characteristics of tourists and environmental elements to redefine and adjust tourism (Higgins-Desbiolles, 2020). The tourism industry should focus on social governance, and effectively prevent and resolve various risks that affect the harmony of the cultural tourism society. For example, the governance mechanism not only creates a safe and stable cultural tourism market environment, but also enhances the people’s sense of gain, happiness and security (Scheyvens et al., 2021).

The role and impact of public opinion WOM has been extensively explored in the literature from the perspective of tourist or consumer behavior. For example, Xie et al. (2020) explored the impact of public opinion climate on tourist risk perception, destination image and tourist satisfaction. Kim et al. (2020) examined tourist perceived performance of smart hotels and exploring its impact on attitude formation and word-of-mouth intentions. Stienmetz et al. (2020) has acknowledged the extent to which perceived P2P accommodation development is associated with changes in community members’ well-being from economic, social, and environmental perspectives. Likewise, we believe that WOMs have a greater impact on individual perceptions than traditional marketer-generated sources of information or even third-party expert reviews. Clearly, individuals make travel decisions based on what they perceive to be the eWOM of their unbiased peers (Nam et al., 2020; Li et al., 2021b). By examining tourists’ word-of-mouth clues in the public opinion and subsequent experiences, we reflect on how the tourism industry should skillfully use public opinion to attract tourists to optimize their travel decisions. However, in the actual travel process, tourists may be affected by their own and environmental elements, and may have multiple perspectives at the same time, which has scarcely been discussed in depth in the existing research. Hence, there is an urgent need to deeply explore the well-being related WOM generated by tourists’ WOM perception under different individual characteristics and environmental elements. Based on the above discussion, this paper explores the impact of tourists’ exposure to eWOM in the context of the social media, focusing on the differences in well-being experience under different individual characteristics and environmental elements. In order to help the tourism industry find a way to make better use of public opinion for social governance, improve the well-being experience of tourists, and thus promote social development.

In summary, first, the current study introduces eWOM perception and livelihood well-being experience as two important independent and dependent variables respectively, and then measures these two indicators in combination with the previous maturity scale. Secondly, individual characteristics such as age and gender as moderator variables with a total of 125 valid questionnaires were examined. We used SPSS software to analyze the data to explore the interaction of eWOM perception, gender, and age on the experience of people’s livelihood and well-being. Finally, with environmental elements as moderator variables, the experiment was divided into three groups: physical environment, social environment and historical environment, and 347 valid questionnaires were obtained to explore the relationship between eWOM perception and people’s well-being experience in different environments.

The aim of this study was to elucidate the intrinsic link between eWOM perception, hedonic well-being, and eudaimonia well-being by exploring causal relationships and regulatory mechanisms. This study also analyzes the moderating effects of tourists’ individual demographics characteristics and environmental elements, confirms the existence and applicability of people’s well-being experience in eWOM, and provides new theoretical insights for the applicable margin of WOM in the field of tourism, which may be conducive to further exploration by researchers to a certain extent. Finally, this study suggests that the tourism industry should continuously promote and optimize online travel service platforms, fully consider the advantages of eWOM dissemination, and combine the personal characteristics and environmental elements of tourists to optimize travelers’ travel strategies and improve people’s travel experience. The well-being of people’s livelihood has certain practical significance for the management of tourism public opinion in social governance.

In order to effectively explore the interaction between tourists’ eWOM perception and individual characteristic elements and environmental elements, this paper designs two studies for empirical analysis. Study 1 selected consumers who booked and stayed at least once in homestays on “Ctrip” as the measurement objects, analyzed the gender and age moderators between eWOM perception and people’s happiness experience, and explored the influence of their interaction with eWOM perception. Individual statistical characteristics of tourists’ hedonic well-being and well-being. In the Study 2, three Xi’an attractions, “Xi’an Ancient City,” “Yuanjia Village” and “Zhongnan Mountain” were selected as experimental materials, to analyze the moderating mechanisms of physical environment, social environment, and historical environment in the relationship between perceptions of eWOM and livelihood well-being experience and then hedonic well-being and eudaimonic well-being.

## Literature review and research hypothesis

### Word-of-mouth perception and livelihood well-being experience

Under the phenomenon of information exchange, word-of-mouth is referred to oral communication between two or more persons about a product or service (Kim et al., 2020), with flexibility, vividness, persuasive, and diagnostic features (Le et al., 2019; Sanchez et al., 2020; Zhao et al., 2020). Specifically, eWOM is easier to obtain and can reach a wider audience faster (Loureiro et al., 2018; Lee and Choi, 2019; Verma and Yadav, 2021). Prior literature has extensively investigated the action mechanism of word of mouth and its electronic form (i.e., eWOM) in the field of hospitality and tourism (Huete-Alcocer, 2017; Yen and Tang, 2019; Pourfakhimi et al., 2020; Zhang et al., 2022), arguing that the perception of word-of-mouth can predict tourists’ behavior (Lee et al., 2022). Therefore, prior scholars have recognized it as an indispensable driving force in the development of tourism (Han et al., 2019).

Previous literature review suggests that tourists’ motivation to use eWOM can be segregated into two aspects. First, it is to build a bridge for effective communication through eWOM, connecting tourists with local residents, service personnel, and the environment (Chen et al., 2018; Jia, 2020; Khalid et al., 2021a). This shapes people’s emotional beliefs when making travel choices so that tourists experience satisfaction in the travel process (Kim et al., 2019). Second, it is the cognitive motivation, said to be an information-intensive network environment. In such an environment, eWOM simplifies formulating a set of decision criteria, reduces uncertainty and risk (Naujoks and Benkenstein, 2020), and promotes the formation of preferences and the differentiation of various alternatives (Hu and Yang, 2020). Moreover, this shapes the cognitive structure and helps consumers increase their decision accuracy, satisfying consumers’ curiosity or information needs (Khalid et al., 2021b; Mainolfi et al., 2022).

In addition to customers satisfaction, well-being is an imperative construct that is referred to as the consumer’s perception of the extent to which a brand (a consumer good or service) contributes positively to various life domains and creates an overall perception of the quality of life, being affected by brands (Hwang and Lee, 2019; Kang, 2020). An Individual’s Well-being experience is segregated into two dimensions: hedonic and eudaimonic well-being (Vada et al., 2019). Hedonic well-being includes positive emotions, happiness, and joy, while eudaimonic well-being focuses on personal growth and self-improvement (Rahmani et al., 2018). Furthermore, hedonic well-being corresponds to effective motivation, as explained above. Likewise, eudaimonic well-being corresponds to cognitive motivation. Hence, customers’ perceived usefulness and enjoyment when they visit a website result in increasing the quality of life in such associated life domains (Kim et al., 2019). Specifically, in the context of online experience, prior findings have linked the value created by customers’ product/service experience with their well-being (Hwang and Lee, 2019; Davlembayeva et al., 2020; Yi et al., 2022). For example, when tourists visit a travel website, they perceive informal interpersonal communication between non-commercial communicators and recipients about brands, products, organizations, or services (Reyes-Menendez et al., 2019), believing that the travel website meets their needs in social and travel life (Choi et al., 2019). That can improve the perceived well-being of various areas of their travel experience and thus enhance their well-being experience (Joseph Sirgy, 2019).

Therefore, this study holds that hedonic well-being and eudaimonic well-being can be enhanced when visitors satisfy their affective and cognitive motivations through perceived positive eWOM. Based on this, this paper proposes the following hypothesis:

*H1*: eWOM perception can significantly and positively influence livelihood well-being experience.

### Moderating effect of individual statistical characteristics elements

Current study on the impact of word of mouth on satisfaction highlights personal characteristics, such as gender (Akinci and Aksoy, 2019; Sun et al., 2019; Craciun et al., 2020) and age (Shaikh et al., 2018; Blanchflower, 2021; Donthu et al., 2021; Zhang et al., 2021), that intend to influence customer behavior. Gender, among other personal characteristics, is a significant reason for individual differences. Prior literature shows that the behavioral differences between men and women are mainly manifested in the following three aspects: first, there are personality differences between men and women (South et al., 2018; Luqman et al., 2022). Such that women are delicate, impulsive, emotional, and easily influenced by others (Li et al., 2019), while men are relatively rational and tend to be more purposeful than women (Ye et al., 2018). Second, men and women communicate socially for different reasons (Long and Tefertiller, 2020). In general, female communication tends to be emotional (Li et al., 2019), preferring to achieve self-emotional enhancement and reciprocity by sharing information with others (Dhir et al., 2016). In contrast, males tend to communicate to exchange information, with a motivation to gain others’ approval (Huber and Malhotra, 2017; Luqman and Zhang, 2022). Third, the level of perceived risk differs between males and females. Moreover, both genders have different attitudes toward cyber technologies (Cai et al., 2017), such that men are more familiar with cyber technologies and perceive less risk as compared to women (Lin X. et al., 2019; Masood et al., 2022).

Furthermore, age is also considered an important predictor of consumer behavior (Li et al., 2021a,b; Wang et al., 2022; Zhou et al., 2023). An individual’s life stages bring variation in their social interaction (i.e., interactions with other individuals, roles, etc.) that is closely related to psychological and physical aging (Josef et al., 2016; Kotter-Grühn et al., 2016; Bodhi et al., 2022). Psychological aging refers to the changes in cognition, personality, and self (Chételat et al., 2018), while physical aging reflects a person’s health status and performance (Michel and Sadana, 2017). Thus, the changes in cognitive abilities, emotional states, physical actions, and age affects consumers’ word-of-mouth perception of a product or service. Consequently, this aids in forming a specific behavior or experience regarding loyalty and well-being (Kim et al., 2019; Luqman et al., 2021; Cuesta-Valino et al., 2022). According to these studies, we believe that in the context of this research, the gender and age of tourists will affect the relationship between their eWOM perception and people’s well-being experience. Hence, we propose the following hypotheses:

*H2a*: Tourist gender moderates the positive relationship between eWOM perception and livelihood well-being experience, such that relationship is strengthened when tourist gender is male vs. female?

*H2b*: Tourist age moderates the positive relationship between eWOM perception and livelihood well-being experience, such that relationship is strengthened when tourist age is low vs. higher?

### Moderating effect of environmental elements

The twin forces of digitalization and globalization have made people’s social and work lives increasingly virtual. This virtual life has left many consumers to feel like those trees that have weak roots and are in danger of being uprooted from the earth (Park, 2019). In response, consumers attempt to (re)connect to places (Ketter and Avraham, 2021), people (van Esch and Mente, 2018), and the past (Hartmann and Brunk, 2019) in order to gain anchor. This is a sense of groundedness (Eichinger et al., 2022) refers to the result of being embedded in physical, social, and historical contexts (Walters et al., 2020). This is concerned with connections between people and places, individual and group identities, and past and present (Cogburn, 2019). First, the sense of groundedness is flourished by connecting with the natural environment or place (Jennings and Bamkole, 2019). This connection can be a literal physical connection, for example, interacting with actual, tangible objects in the local environment or being immersed in the natural environment (Cai et al., 2020; Saleem et al., 2021). In addition to physical connections, tourists can also connect with more symbolic places, for example, by consuming local products or services during the course of a trip, thus creating a connection with the destination (Li and Katsumata, 2020; Wondirad et al., 2021). Second, the sense of groundedness can be formed by connecting to the social environment (Borghi et al., 2019). This connection can be achieved not only through the community creating a familiar atmosphere of family (Wu et al., 2018). However, it is also shaped by visitors who communicate with locals (Lin P. M. et al., 2019), emphasizing a sense of belonging to an identity (Hung et al., 2019). Finally, the visitors’ connection with the past, i.e., the historical environment, can shape the groundedness (Su et al., 2020; Shahbaz et al., 2021). Tourism destinations can create nostalgic brands through historical stories’ narratives and cultural resource development, providing tourists with the basis of memory, tradition, and cultural values (O'Connor, 2022). Tourists are also inspired to dwell on the past, make the historic scenes live again, and help tourists understand the roots of tourist destinations (Mun et al., 2018). Thus, a sense of groundedness can serve as a foundation for social and individual well-being based on people’s connection to their physical, social, and historical environments, giving people strength, security, and stability (Yang et al., 2020).

Drawing from prior findings, we argue that eWOM, as an external environmental stimulus, allows people to establish an emotional connection with their physical, social, and historical environment, providing a sense of balance, belongingness, and thus enhancing their experience of well-being. Hence, we propose the following hypotheses:

*H3a*: The physical environment significantly moderates the positive relationship between eWOM perception and livelihood well-being experience.

*H3b*: The social environment significantly moderates the positive relationship between eWOM perception and livelihood well-being experience.

*H3c*: The historical environment significantly moderates the positive relationship between eWOM perception and livelihood well-being experience.

**Figure 1:**
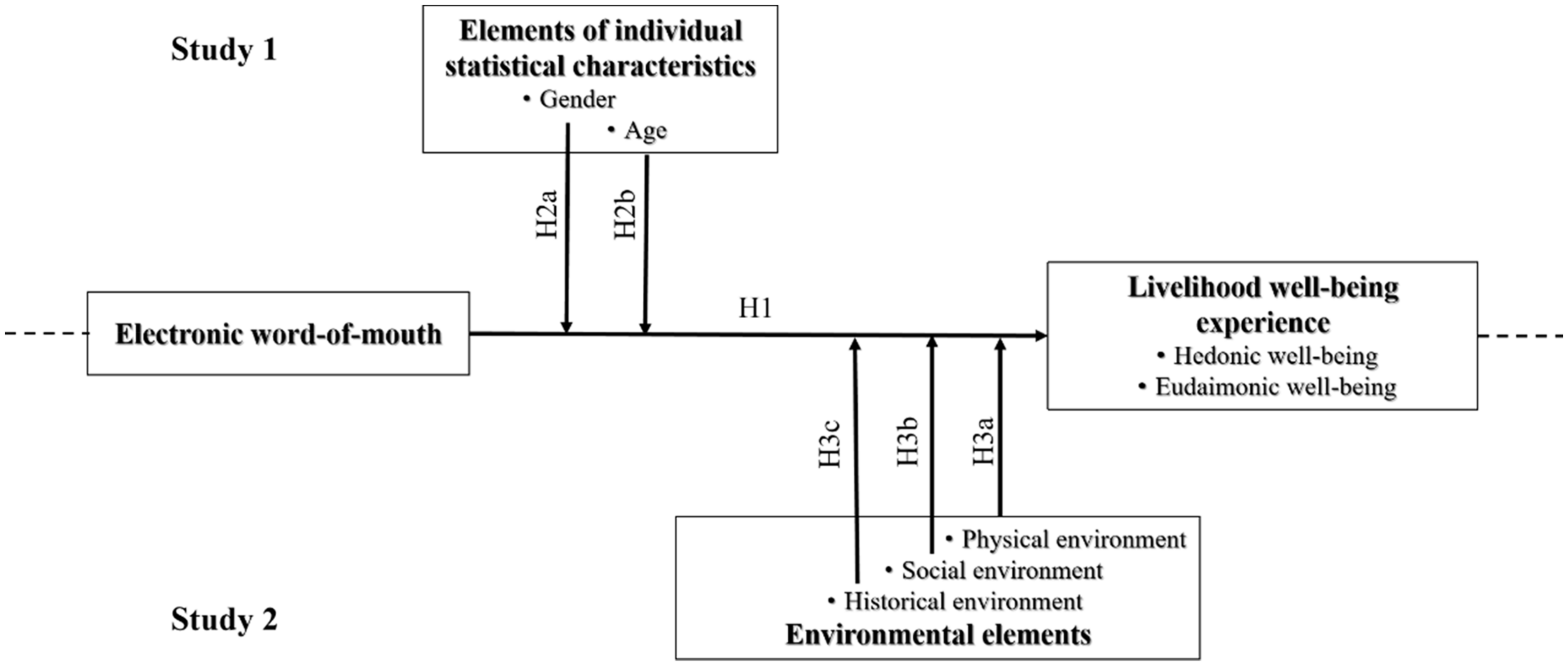
Theoretical model diagram.

Combining hypotheses H1, H2a, H2b, H3a, H3b and H3c, the corresponding theoretical model is plotted, as shown in Figure 1. The upper part of the model diagram is the content of Sub-study 1, that is, to verify the correlation between eWOM perception and people’s well-being experience, and the moderating role of different individual characteristics (i.e., gender and age) in it. The lower part of the model diagram is the content of Sub-study 2, that is, to re-verify the correlation between eWOM perception and people’s livelihood and well-being experience, and explore the moderating role of different environmental elements (i.e., physical environment, social environment and historical environment) in it.

## Research design

To explore the value of tourism public opinion management in social governance, this study investigates its effects on livelihood well-being experience (i.e., hedonic well-being and eudaimonic well-being) from the perspective of tourists’ eWOM perception in the context of internet era. The tourist’s eWOM perception, directly affecting peoples’ livelihood well-being, may also be influenced by personal statistical characteristics and environmental elements. Therefore, this study introduces personal statistical characteristics and environmental elements as moderating variables. These moderating variables help better explain the relationship between eWOM and people’s livelihood well-being experience. This study considered personal statistical characteristics in terms of gender, i.e., male and female, and age measured in three groups, i.e., youth (18–40 years), middle age (41–64 years), and old age (≥65 years). In addition, we considered environmental elements in terms of the physical, social, and historical environment. We adapted the measurement items from the maturity scale with slight amendments according to this study’s context. These measurement items encompass five questions on eWOM perception (Set them to KB1-KB5) from Jalilvand and Samiei's (2012) eWOM scale and eight questions on livelihood well-being experience from Vada et al.’s (2019) well-being scale. The livelihood well-being experience consists of five questions on hedonic well-being (Set them to XL1–XL5) and three questions on eudaimonic well-being (Set them to SX1–SX3).

Two studies are designed in the current paper considering the above measurements. Whereby, study 1 analyzes the moderating mechanisms of personal characteristics (i.e., gender and age) between eWOM perceptions and livelihood well-being experience, and investigates the interaction affect between eWOM perceptions and individual statistical characteristics of tourists on hedonic well-being and eudaimonic well-being. Study 2 analyzes the moderating mechanisms of the physical environment, social environment, and historical environment in the relationship between eWOM perceptions and livelihood well-being experience and explores the interaction effect between eWOM perceptions and environmental elements on hedonic well-being and eudaimonic well-being, respectively.

## Sub-study 1: Moderating mechanisms of the elements of individual statistical characteristics

At present, many travelers prefer to book B&B (Bed and Breakfast) services for the short term to better interact with locals and experience their lifestyles. Several other platforms like this provide relatively easy and safe conditions for such interactions. Among all such platforms, Ctrip is renowned for providing a mapping mechanism to match the services for hosts and customers. Furthermore, Ctrip is the world’s leading travel service contact center, which provides quality travel options and guarantees standardization and service quality. Therefore, we considered “Ctrip” as the material for this study to measure the subjects. First, those consumers who booked on “Ctrip” and checked in at least once were selected as measurement objects. Next, we collected the demographic information of those consumers. Many prior studies have shown the significant impact of gender and age on consumer behavior and are important criteria for consumer classification to be analyzed (Pícha and Navrátil, 2019; Moon, 2021; Nusrat et al., 2021; Zhang et al., 2021). Thus, these are considered as moderating variables. Finally, we use SPSS to analyze the related data to test the moderating effect of individual characteristics on eWOM perception and people’s well-being experience (hedonic happiness and eudaimonic happiness) when using “Ctrip.” After calculating the average value of eWOM perception scores, the data can be divided into two categories, i.e., 0 = low eWOM perception (≤3), and 1 = high eWOM perception (>3), for further processing. Moreover, the items of the samples’ hedonic happiness and eudaimonic happiness are scored on a scale of 1–5. The final calculated averages represent hedonic happiness and eudaimonic happiness. After excluding invalid questionnaires, this study considered 125 valid samples, including 66 males (52.8%) and 59 females (47.2%). The basic statistics of each variable are presented in Table 1. We used SPSS multivariate ANOVA to test the moderating mechanism of gender and age, as shown in the analysis in sections Moderating mechanisms of gender and Moderating mechanisms of age, Moderating mechanisms of the social environment, and Moderating mechanisms of the historical environment.

**Table 1:** Study 1: Descriptive statistical analysis of each variable.

	Mean	Median	Min	Max	S.D.	Kurtosis	Skewness
KB1	2.92	3	1	5	1.707	−1.714	0.097
KB2	3.02	3	1	5	1.626	−1.629	−0.072
KB3	2.94	3	1	5	1.664	−1.692	0.039
KB4	3.02	3	1	5	1.675	−1.682	0.006
KB5	2.94	3	1	5	1.677	−1.690	0.017
XL1	3.00	3	1	5	1.704	−1.688	0.010
XL2	2.99	3	1	5	1.521	−1.482	0.042
XL3	2.80	3	1	5	1.476	−1.375	0.184
XL4	2.98	3	1	5	1.677	−1.712	0.049
XL5	2.90	2	1	5	1.701	−1.738	0.092
SX1	2.78	3	1	5	1.616	−1.604	0.138
SX2	2.81	2	1	5	1.610	−1.608	0.177
SX3	2.76	2	1	5	1.603	−1.593	0.209

### Moderating mechanisms of gender

#### Results

This study found a significant impact of eWOM perception (low and high) on hedonic well-being (M_low_ = 1.539, SD = 0.376; M_high_ = 4.447, SD = 0.495, *p* < 0.001). While the main effect of tourist gender identity (male and female) on hedonic well-being was also significant (M_male_ = 2.767, SD = 1.427; M_female_ = 3.122, SD = 1.614, *p* < 0.001). The study results showed the significant interaction of eWOM perception and tourist gender identity on hedonic well-being (*F* = 11.321, *p* = 0.001). Meanwhile, the main effect of eWOM perception (low and high) on eudaimonic well-being was also significant (M_low_ = 1.369, SD = 0.334; M_high_ = 4.311, SD = 0.531, *p* < 0.001). We further found that tourist gender identity (male and female) significantly influences the eudaimonic well-being (M_male_ = 2.970, SD = 1.698; M_female_ = 2.571, SD = 1.322, *p* < 0.001) with the significant interaction of eWOM perception and tourist gender identity on eudaimonic well-being (*F* = 35.491, *p* < 0.001).

**Figure 2:**
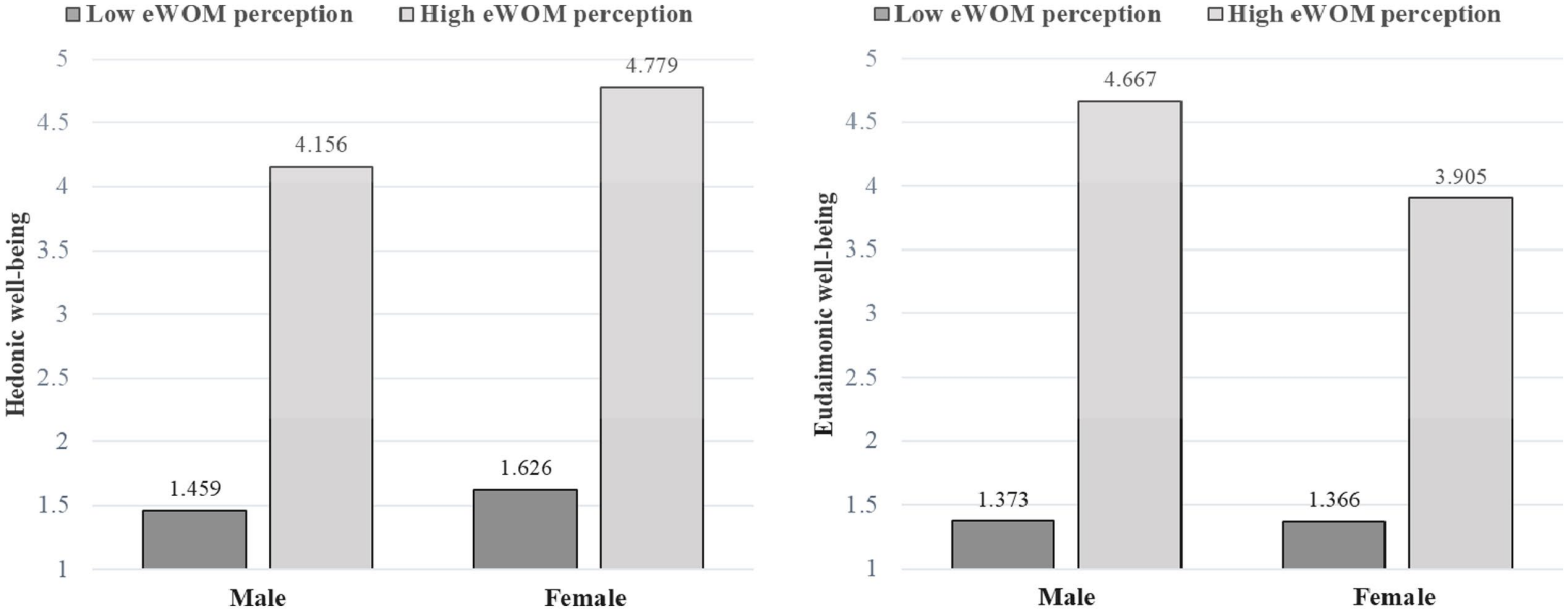
Interaction between eWOM perception and gender identity.

Simple effects analysis (as in Figure 2) revealed that in the case of males, the hedonic well-being developed by high eWOM perception (M_high_ = 4.156, SD = 0.500) was significantly higher than that of low eWOM perception (M_low_ = 1.459, SD = 0.377, *p* < 0.05). Similarly, in the case of females, the hedonic well-being developed by high eWOM perception (M_high_ = 4.779, SD = 0.183) was also significantly higher than that of low eWOM perceptions (M_low_ = 1.626, SD = 0.361, *p* < 0.05). At the same time, for males, the eudaimonic well-being developed by high eWOM perceptions (M_high_ = 4.667, SD = 0.369) was significantly higher than that of low eWOM perceptions (M_low_ = 1.373, SD = 0.365, *p* < 0.05). Furthermore, the eudaimonic well-being developed by high eWOM perceptions (M_high_ = 3.905, SD = 0.372) was significantly higher than that of low eWOM perceptions (M_low_ = 1.366, SD = 0.303, *p* < 0.05). Hence, we can observe the interaction between eWOM perceptions and tourist gender identity on hedonic and eudaimonic well-being is significant.

#### Discussion

Initially, this section found a significant positive effect of eWOM perception on livelihood well-being experience (hedonic well-being and eudaimonic well-being), which verified hypothesis H1. In addition, we discovered the significant differences in the interaction effect between eWOM perception and tourist gender identity on hedonic well-being and eudaimonic well-being. For female tourists, hedonic well-being was higher for high eWOM perception (vs. low eWOM perception). On the other hand, eudaimonic well-being was higher for male-identified tourists. Hence, these results verified hypothesis H2a.

However, for moderating effect of age, the following research explains the effect of the interaction between eWOM perception and the age group of the tourists on hedonic and eudaimonic well-being.

### Moderating mechanisms of age

#### Results

This study investigated the moderation effect of age and found a significant impact of tourists’ age group (youth, middle age, and old age) on hedonic well-being (M_youth_ = 3.956, SD = 0.732; M_middle age_ = 1.672, SD = 0.703; M_old age_ = 4.500, SD = 0.933, *p* = 0.005). Furthermore, the interaction effect between eWOM perception and tourists’ age group on hedonic well-being was also significant (*F* = 5.165, *p* = 0.007). This study results also found the main significant effect of tourists’ age group (youth, middle age, and old age) on eudaimonic well-being (M_youth_ = 4.593, SD = 0.869; M_middle age_ = 1.490, SD = 0.732; M_old age_ = 3.775, SD = 0.590, *p* < 0.001). The interaction effect between eWOM perception and tourists’ age group on eudaimonic well-being was also significant (*F* = 12.425, *p* < 0.001).

**Figure 3:**
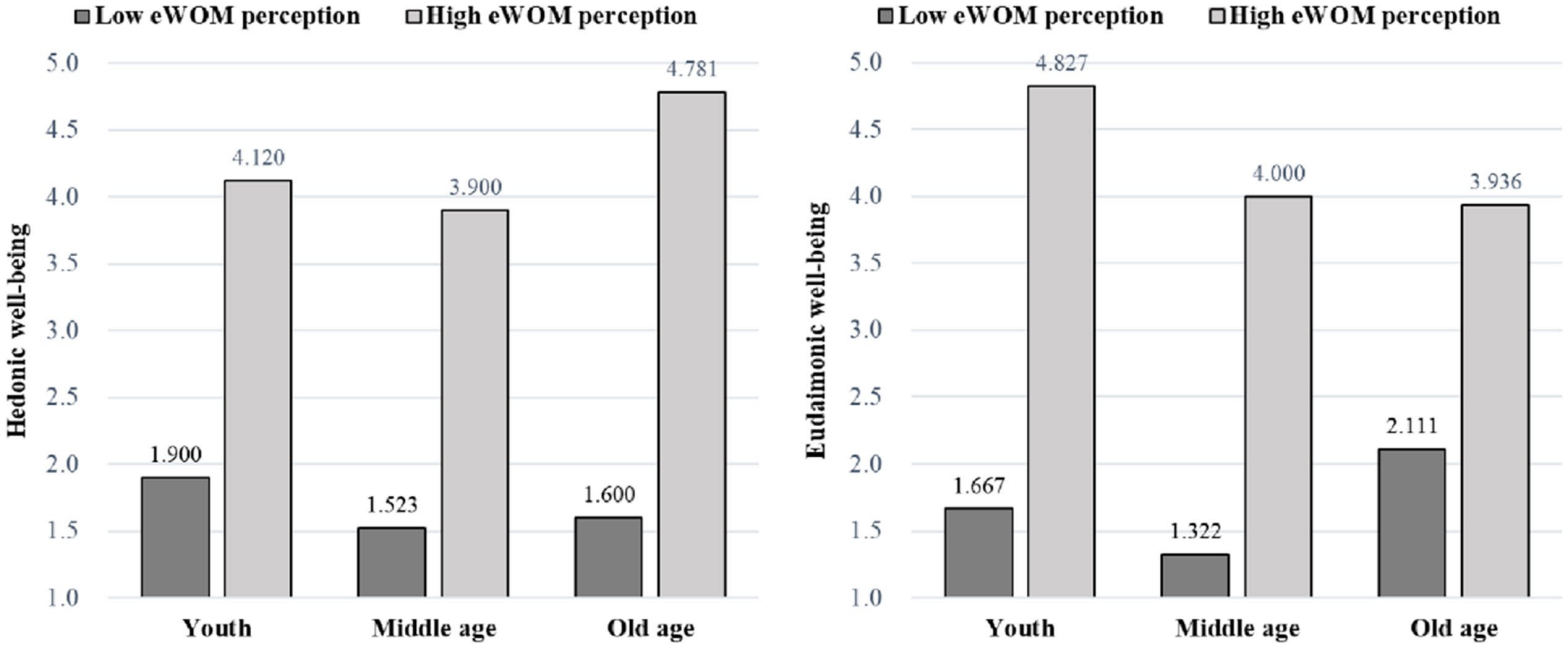
Interaction between eWOM perception and age group.

The study results (as in Figure 3) revealed that for young people, the hedonic well-being formed by the high eWOM perception (M_high_ = 4.120, SD = 0.424) was significantly higher than that of the low eWOM perception (M_low_ = 1.900, SD = 0.707, *p* < 0.05). While for middle aged, hedonic well-being developed by high eWOM perception (M_high_ = 3.900, SD = 0.757) was significantly higher than that of low eWOM perceptions (M_low_ = 1.523, SD = 0.374, *p* < 0.05). The results further showed for elderly people, hedonic well-being developed by high eWOM perceptions (M_high_ = 4.781, SD = 0.189) was significantly higher than that of low eWOM perceptions (M_low_ = 1.600, SD =0.000, *p* < 0.05). Furthermore, in case of eudaimonic well-being, young people showed significant eudaimonic well-being formed by high eWOM perception (M_high_ = 4.827, SD = 0.218) than that of low eWOM perception (M_low_ = 1.667, SD = 0.000, *p* < 0.05). While for middle aged, the eudaimonic well-being formed by high eWOM perception (M_high_ = 4.000, SD = 0.770) was significantly higher than that of low eWOM perceptions (M_low_ = 1.322, SD = 0.294, p < 0.05). Similarly, for elderly people, the eudaimonic well-being developed by high eWOM perceptions (M_high_ = 3.936, SD = 0.278) was significantly higher than that of low eWOM perceptions (M_low_ = 2.111, SD = 0.192, *p* < 0.05). Thus, this study observed the interaction between eWOM perception and the age of tourists on hedonic well-being and eudaimonic well-being is significant.

#### Discussion

This section found a significant positive effect of eWOM perception on livelihood well-being experience (hedonic well-being and eudaimonic well-being), which verified hypothesis H1. In addition, we discovered a significant difference in the interaction effect between eWOM perception and the tourists’ age group on hedonic and eudaimonic well-being. In the high eWOM perception, elderly tourists have the highest hedonic happiness, followed by young tourists. While middle-aged tourists experience the lowest hedonic happiness. For young people in high eWOM perception, they experience the highest eudaimonic happiness, followed by middle-aged tourists. While older tourists have the lowest eudaimonic happiness. Hence, these results verified hypothesis H2b.

However, the relationship between eWOM perception and well-being experience can be also influenced by different environments. The impact of the interaction of eWOM perception and environmental elements on the experience of livelihood well-being has been explored in the study 2.

## Sub-study 2: Moderating mechanisms of environmental elements

With today’s tremendous tourism development, Xi’an has become a popular city and well-known over the internet due to its rich tourism resources and internet publicity. While Xi’an has made every effort to ensure the level of tourism services. This city has also created a wonderful cultural and tourism feast for tourists from all over the country. That includes an immersive cultural experiences, folklore and cultural activities, camping and other cultural and tourism activities for the people. To further fit the context of the physical, social, and historical environment, three Xi’an attractions, namely “Xi’an Old Town,” “Yuanjia Village,” and “Mount Zhongnan,” were selected as the study material. The subjects were divided into three groups for measurement.

**Figure 4:**
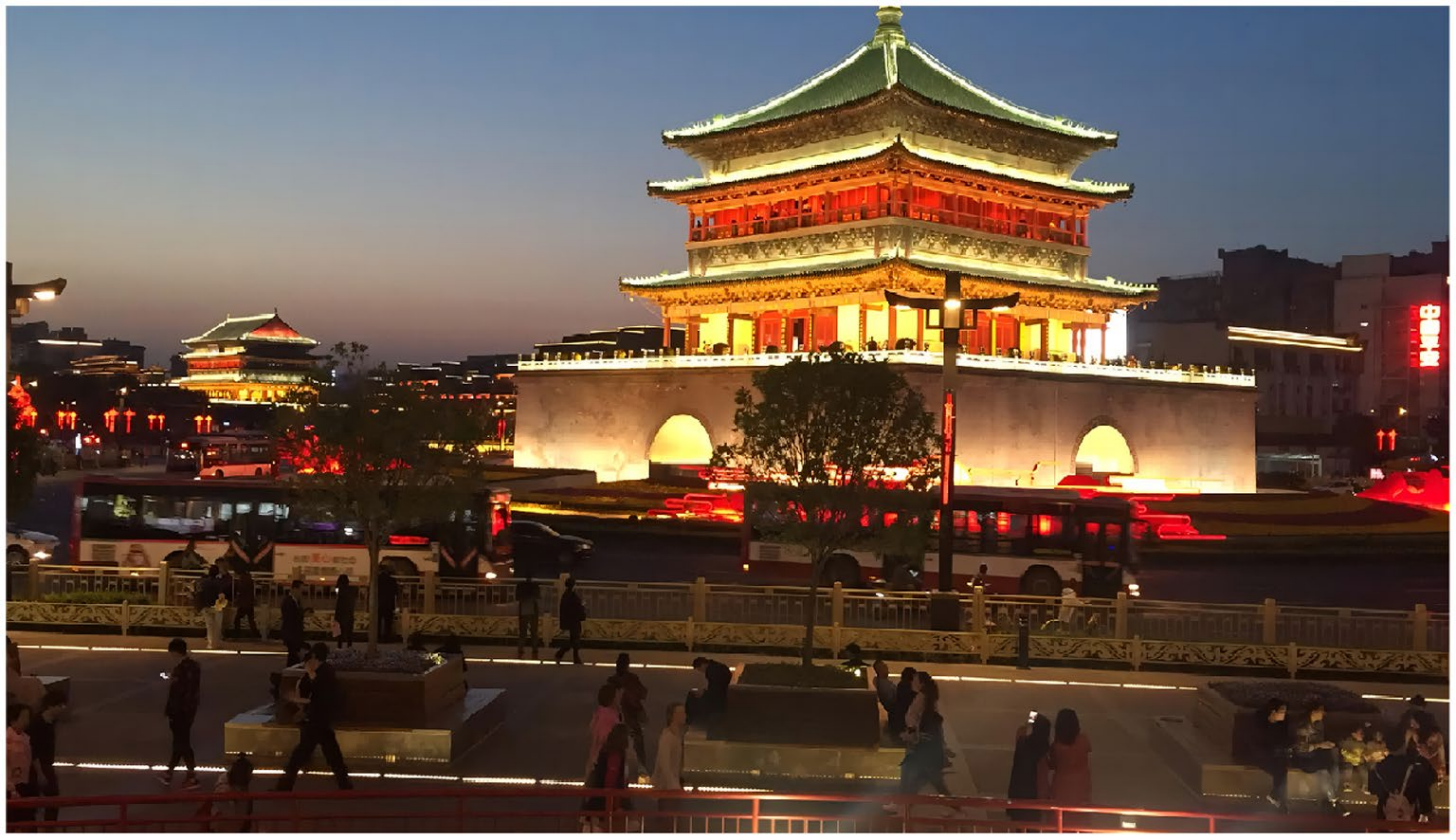
Xi’an old town (physical environment).

**Figure 5:**
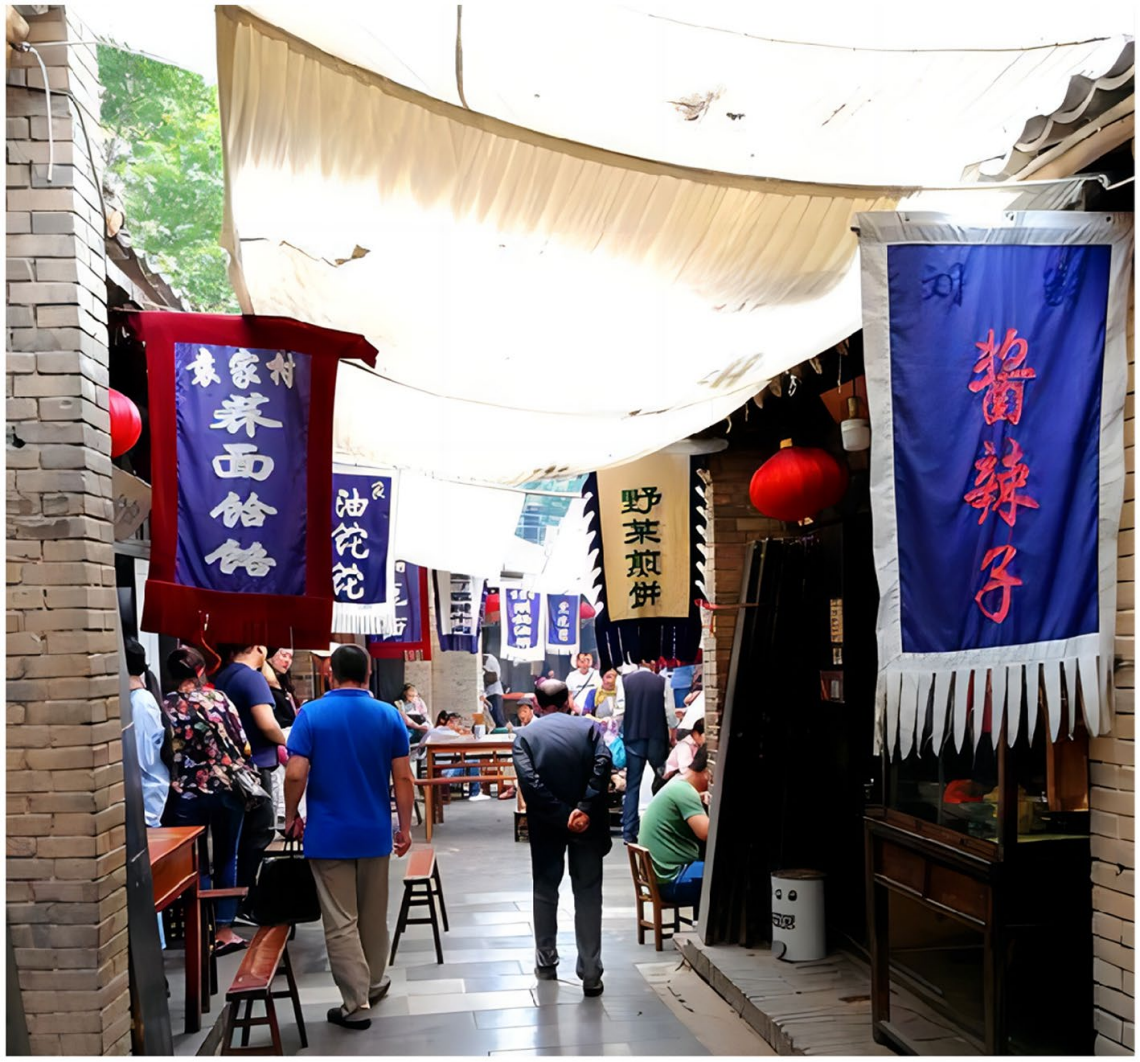
Yuanjia village (social environment).

**Figure 6:**
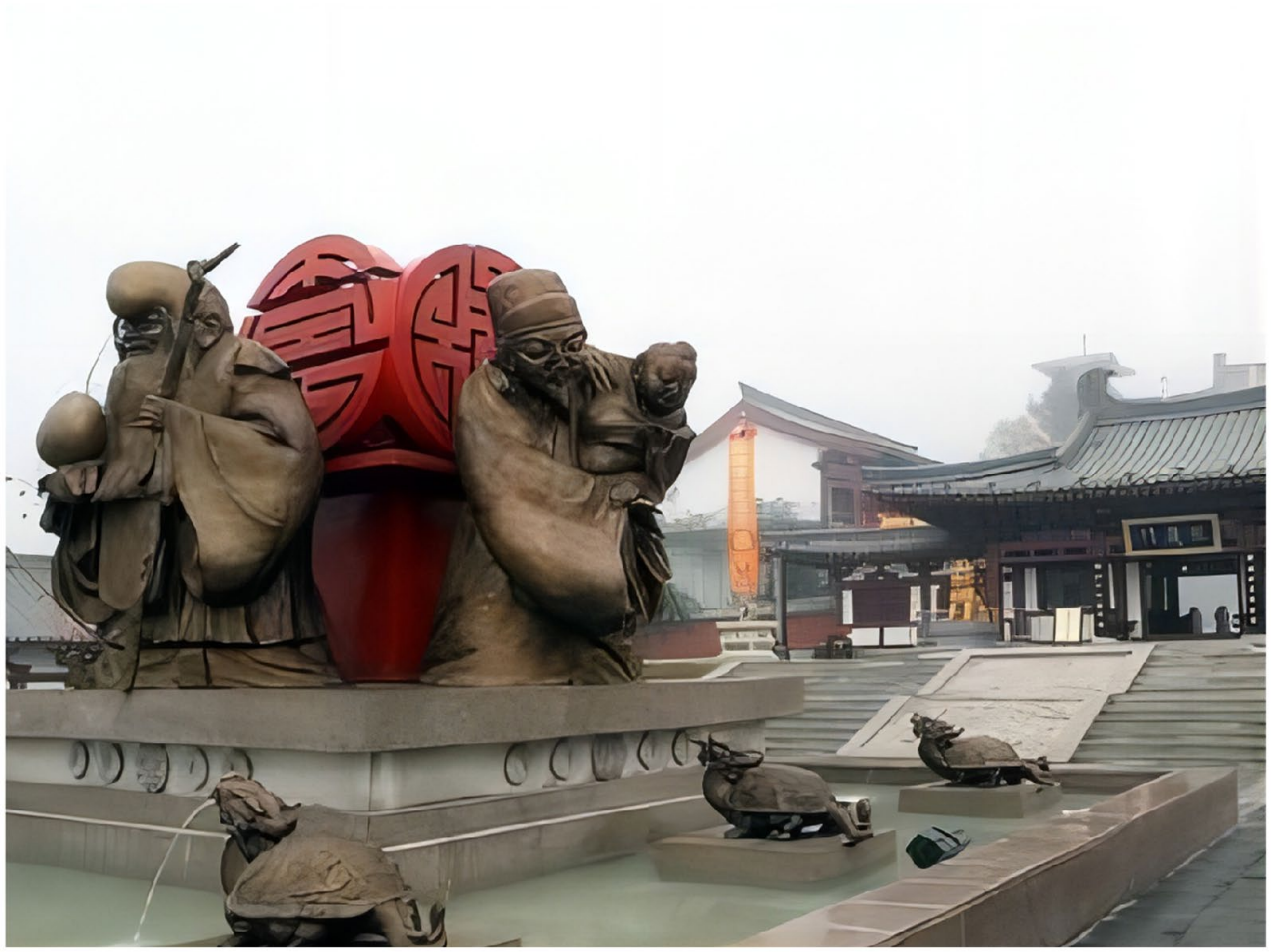
Mount Zhongnan (historical environment).

The experiment in study 2 let the subjects imagine that they had seen the relevant online reviews on “Ctrip,” accompanied by corresponding pictures. The first group was the physical environment test group (as shown in Figure 4). The comment was “on the wall of the ancient city, looking at the Bell and Drum Tower, the Big Wild Goose Pagoda and the Small Wild Goose Pagoda from afar, and overlooking the streets and alleys of the ancient city, tasting the changes of the ancient city.” In this comment, the words “from afar” and “overlooking” were used to reflect the sense of physical space. Next, the second group in the study was the social environment test group (as shown in Figure 5). The comment was “going to Yuanjia Village to communicate with the locals, tasting the food and experiencing the rural culture.” In this comment the words such as “communicate” and “tasting” were used to reflect a kind of interpersonal atmosphere. Lastly, the third group was the historical environment test group (as shown in Figure 6). The comment was “going to Mount Zhongnan to pursue traditional cultures such as ‘Taoist Culture’, ‘Filial Piety Culture’, ‘Longevity Culture’, and ‘God of Wealth Culture’, which are most admired by the people, and appreciating the thousands of years of literati and writers who have left a large number of Cultural relics, precious calligraphy, etc.” in this comment the words such as “traditional cultures,” “thousands of years” and “relics” were used to reflect a kind of memory perception of history. Moving on, the subjects were asked to fill in three items on the perception of environmental elements (Set the items of the physical environment to WL1-WL3, the items of the social environment to SH1-SH3, and the items of historical environment to LS1-LS3). Then, after eliminating the invalid responses, we obtained 347 valid samples were obtained, and the three experimental groups were set to 115, 119, and 113 people, respectively. Finally, we used SmartPLS software to test the relationship between the participants’ perception of eWOM and the interaction of environmental elements and the people’s well-being experience (hedonic well-being and eudaimonic well-being). The moderating mechanisms of physical context, social context perception, and historical context analysis were then tested using PLS-SEM, as shown in the analyses in sections Moderating mechanisms of the physical environment, Moderating mechanisms of the social environment, and Moderating mechanisms of the historical environment.

### Moderating mechanisms of the physical environment

#### Results

To explore the moderating mechanism of the physical environment, we first performed a descriptive statistical analysis of all variables, as shown in Table 2. It suggests that the mean value corresponding to each variable is about 3, and the standard deviation is within 3, indicating that there are no outliers in each variable, which can be preliminarily judged to be normal distribution. Second, the standardized results were used for the analysis (shown in Table 3). As for eWOM perception, its Cronbach’s Alpha is 0.983, composite reliability (CR) is 0.986, and average extracted variance (AVE) is 0.936; as for hedonic well-being, its Cronbach’s Alpha is 0.970, composite reliability (CR) is 0.977, and average extracted variance (AVE) is 0.895; as for eudaimonic well-being, its Cronbach’s Alpha is 0.924, composite reliability (CR) is 0.952, and average extracted variance (AVE) is 0.869; as for physical environment, its Cronbach’s Alpha is 0.914, composite reliability (CR) is 0.946, and average extracted variance (AVE) is 0.854. Following the existing studies (e.g., Gong et al., 2020; Luqman et al., 2020a,b), We found that Cronbach’s Alpha coefficients of eWOM perception, hedonic well-being, eudaimonic well-being and physical environment were all greater than 0.8. Moreover, the composite reliability (CR) was greater than 0.8, and the average extracted variation (AVE) was greater than 0.5. These results indicated that each variable has a good convergent validity. Finally, we used bootstrapping method to calculate each path coefficient and the corresponding T-statistic and value of *p* (as shown in Table 4). We found that eWOM perception had a significant positive effect on both hedonic and eudaimonic well-being (β _hedonic_ = 0.514, *p* < 0.001; β _eudaimonic_ = 0.644, *p* < 0.001). Furthermore, the eWOM perception and physical environment interaction on hedonic and eudaimonic well-being was also found significant (T _hedonic_ = 3.892, p < 0.001; T _eudaimonic_ = 2.244, *p* = 0.025).

**Table 2:** Study 2: Descriptive statistical analysis of each variable (physical environment).

	Mean	Median	Min	Max	S.D.	Kurtosis	Skewness
KB1	3.365	4	1	5	1.41	−0.98	−0.729
KB2	3.278	4	1	5	1.552	−1.278	−0.659
KB3	3.4	4	1	5	1.437	−1.008	−0.713
KB4	3.522	4	1	5	1.716	−1.346	−0.691
KB5	3.73	5	1	5	1.716	−1.202	−0.818
WL1	3.409	4	1	5	1.636	−1.449	−0.427
WL2	3.235	3	1	5	1.545	−1.383	−0.243
WL3	2.87	3	1	5	1.289	−0.871	−0.049
XL1	3.357	4	1	5	1.611	−1.307	−0.648
XL2	3.652	4	1	5	1.549	−1.084	−0.751
XL3	3.53	4	1	5	1.721	−1.347	−0.695
XL4	2.843	3	1	5	1.412	−1.117	0
XL5	2.878	3	1	5	1.452	−1.168	0.025
SX1	3.113	3	1	5	1.443	−1.215	−0.13
SX2	3.539	4	1	5	1.68	−1.41	−0.57
SX3	3.4	4	1	5	1.525	−1.147	−0.686

**Table 3:** Study 2: The facet reliability and validity of each variable (physical environment).

	Cronbach’s alpha	rho_A	CR	AVE
eWOM perception	0.983	0.983	0.986	0.936
Hedonic well-being	0.97	0.971	0.977	0.895
Eudaimonic well-being	0.924	0.926	0.952	0.869
Physical environment	0.914	0.915	0.946	0.854

**Table 4:** Study 2: Path Coefficients under moderating effect (physical environment).

	Original sample (O)	Sample mean (M)	Standard deviation (STDEV)	T statistic (|O/STDEV|)	*p*-value
eWOM perception → Hedonic well-being	0.514	0.511	0.028	18.573	0.000
eWOM perception → Eudaimonic well-being	0.644	0.648	0.050	12.831	0.000
Moderating effect 1 → Hedonic well-being	0.075	0.073	0.019	3.892	0.000
Moderating effect 2 → Eudaimonic well-being	0.090	0.091	0.040	2.244	0.025

#### Discussion

This section found a significant positive effect of eWOM perception on hedonic well-being and eudaimonic well-being. Thus, hypothesis H1 was verified. And we also preliminarily concluded the significant moderating effect of physical environment in the relationship between eWOM perception and hedonic and eudaimonic well-being. The study findings indicated that tourists with a high level of physical environment would have more positive effects on their livelihood well-being experience with high eWOM perception, as compared to low level of the physical environment. Therefore, hypothesis H3a was verified.

However, we explored the moderating effect of social environment between eWOM perception and hedonic and eudaimonic well-being in the next study.

### Moderating mechanisms of the social environment

#### Results

We first performed a descriptive statistical analysis considering all the study variable (shown in Table 5), which suggested that the mean value corresponding to each variable was about 3, and the standard deviation was within 3, indicating that there were no outliers in each variable. Second, using standardized results for analysis (shown in Table 6), we obtained Cronbach’s alpha coefficients greater than 0.8 for eWOM perception, hedonic well-being, eudaimonic well-being, and social environment. Next, we found the reliability (CR) results greater than 0.8, and the average extracted variance (AVE) was greater than 0.5. As for eWOM perception, its Cronbach’s Alpha is 0.981, composite reliability (CR) is 0.985, and average extracted variance (AVE) is 0.930; as for hedonic well-being, its Cronbach’s Alpha is 0.967, composite reliability (CR) is 0.975, and average extracted variance (AVE) is 0.885; as for eudaimonic well-being, its Cronbach’s Alpha is 0.922, composite reliability (CR) is 0.951, and average extracted variance (AVE) is 0.865; as for social environment, its Cronbach’s Alpha is 0.907, composite reliability (CR) is 0.942, and average extracted variance (AVE) is 0.844. These results indicated a good convergent validity for each variable. Finally, we used Bootstrapping method to calculate each path coefficient and the corresponding T-statistic and value of p (as shown in Table 7). We found that eWOM perception had a significant positive effect on both hedonic and eudaimonic well-being (β _hedonic_ = 0.516, *p* < 0.001; β _eudaimonic_ = 0.674, *p* < 0.001). Moreover, the eWOM perception and social environment interaction on hedonic and eudaimonic well-being were also significant (T _hedonic_ = 3.295, *p* = 0.001; T _eudaimonic_ = 2.762, *p* = 0.006).

**Table 5:** Study 2: Descriptive statistical analysis of each variable (social environment).

	Mean	Median	Min	Max	S.D.	Kurtosis	Skewness
KB1	3.454	4	1	5	1.407	−0.792	−0.831
KB2	3.387	4	1	5	1.529	−1.064	−0.775
KB3	3.487	4	1	5	1.431	−0.821	−0.819
KB4	3.605	4	1	5	1.666	−1.125	−0.81
KB5	3.807	5	1	5	1.657	−0.949	−0.934
SH1	3.479	4	1	5	1.587	−1.301	−0.517
SH2	3.311	3	1	5	1.505	−1.269	−0.335
SH3	2.941	3	1	5	1.259	−0.784	−0.144
XL1	3.437	4	1	5	1.565	−1.087	−0.768
XL2	3.723	4	1	5	1.489	−0.836	−0.858
XL3	3.605	4	1	5	1.666	−1.125	−0.81
XL4	2.916	3	1	5	1.382	−1.047	−0.099
XL5	2.983	3	1	5	1.455	−1.157	−0.053
SX1	3.168	3	1	5	1.404	−1.11	−0.23
SX2	3.605	4	1	5	1.626	−1.237	−0.66
SX3	3.521	4	1	5	1.505	−0.931	−0.804

**Table 6:** Study 2: The facet reliability and validity of each variable (social environment).

	Cronbach’s alpha	rho_A	CR	AVE
eWOM perception	0.981	0.982	0.985	0.93
Hedonic well-being	0.967	0.968	0.975	0.885
Eudaimonic well-being	0.922	0.923	0.951	0.865
Social environment	0.907	0.908	0.942	0.844

**Table 7:** Study 2: Path coefficients under moderating effect (social environment).

	Original sample (O)	Sample mean (M)	Standard deviation (STDEV)	T statistic (|O/STDEV|)	*P*-value
eWOM perception → Hedonic well-being	0.516	0.512	0.031	16.916	0.000
eWOM → Eudaimonic well-being	0.674	0.676	0.052	13.079	0.000
Moderating effect 1 → Hedonic well-being	0.067	0.064	0.020	3.295	0.001
Moderating effect 2 → Eudaimonic well-being	0.110	0.111	0.040	2.762	0.006

#### Discussion

This section found a significant positive effect of eWOM perception on hedonic well-being and eudaimonic well-being. Thus, hypothesis H1 was verified. And we also concluded the significant moderating effect of social environment between eWOM perception and hedonic well-being and eudaimonic well-being. This study observed that high eWOM perceptions have more positive effects on tourists’ livelihood well-being experience in a high-level social environment compared to a low-level social environment. Therefore, hypothesis H3b was verified.

However, since the environmental element of this study also includes the historical environment, thus, the following research will focus on the moderating effect of the historical environment between eWOM perception and hedonic and eudaimonic well-being.

### Moderating mechanisms of the historical environment

#### Results

Similar to the previous studies, we first performed a descriptive statistical analysis of all variables in this study, as shown in Table 8. Second, using standardized results for analysis, we obtained Cronbach’s alpha coefficients greater than 0.8 for eWOM perception (shown in Table 9), hedonic well-being, eudaimonic well-being, and historical environment. We also found the reliability (CR) greater than 0.8, and the average extracted variance (AVE) was greater than 0.5. Among them, the Cronbach’s Alpha of eWOM perception is 0.976, composite reliability (CR) is 0.981, and average extracted variance (AVE) is 0.912; the Cronbach’s Alpha of hedonic well-being is 0.959, composite reliability (CR) is 0.968, and average extracted variance (AVE) is 0.858; the Cronbach’s Alpha of eudaimonic well-being is 0.909, composite reliability (CR) is 0.943, and average extracted variance (AVE) is 0.846; the Cronbach’s Alpha of historical environment is 0.887, composite reliability (CR) is 0.931, and average extracted variance (AVE) is 0.818. These results revealed a good convergent validity for each variable. Finally, we used Bootstrapping method to calculate each path coefficient and the corresponding T-statistic and value of *p* (as shown in Table 10). We found that eWOM perception had a significant positive effect on both hedonic and eudaimonic well-being (β _hedonic_ = 0.544, *p* < 0.001; β _eudaimonic_ = 0.705, *p* < 0.001). Moreover, the eWOM perception and historical environment interaction on hedonic and eudaimonic well-being were also significant (T _hedonic_ = 2.825, *p* = 0.005; T _eudaimonic_ = 2.801, *p* = 0.005).

**Table 8:** Study 2: Descriptive statistical analysis of each variable (historical environment).

	Mean	Median	Min	Max	S.D.	Kurtosis	Skewness
KB1	3.655	4	1	5	1.309	0.019	−1.136
KB2	3.619	4	1	5	1.422	−0.287	−1.096
KB3	3.699	4	1	5	1.336	−0.035	−1.123
KB4	3.832	4	1	5	1.534	−0.336	−1.145
KB5	4.018	5	1	5	1.517	−0.07	−1.28
LS1	3.673	4	1	5	1.484	−0.854	−0.753
LS2	3.496	4	1	5	1.415	−0.933	−0.532
LS3	3.106	3	1	5	1.193	−0.468	−0.303
XL1	3.646	4	1	5	1.439	−0.305	−1.093
XL2	3.938	4	1	5	1.378	−0.021	−1.183
XL3	3.841	4	1	5	1.538	−0.333	−1.15
XL4	3.124	3	1	5	1.338	−0.821	−0.252
XL5	3.195	3	1	5	1.407	−0.982	−0.217
SX1	3.327	3	1	5	1.327	−0.801	−0.416
SX2	3.805	5	1	5	1.51	−0.674	−0.929
SX3	3.743	4	1	5	1.4	−0.152	−1.119

**Table 9:** Study 2: The facet reliability and validity of each variable (historical environment).

	Cronbach’s alpha	rho_A	CR	AVE
eWOM perception	0.976	0.977	0.981	0.912
Hedonic well-being	0.959	0.959	0.968	0.858
Eudaimonic well-being	0.909	0.91	0.943	0.846
Historical environment	0.887	0.889	0.931	0.818

**Table 10:** Study 2: Path coefficients under moderating effect (historical environment).

	Original sample (O)	Sample mean (M)	Standard deviation (STDEV)	T statistic (|O/STDEV|)	P-value
eWOM perception → Hedonic well-being	0.544	0.539	0.037	14.784	0.000
eWOM perception → Eudaimonic well-being	0.705	0.711	0.067	10.505	0.000
Moderating effect 1 → Hedonic well-being	0.061	0.059	0.022	2.825	0.005
Moderating effect 2 → Eudaimonic well-being	0.116	0.118	0.041	2.801	0.005

#### Discussion

This part discovered that eWOM perceptions had a significant positive effect on hedonic well-being and eudaimonic well-being. Thus, hypothesis H1 was verified. The results also verified the significant moderating effect of historical environment in the relationship between eWOM perception and hedonic well-being and eudaimonic well-being. Moreover, a high level of historical environment has a more positive effect on tourists’ livelihood well-being experience with high eWOM perceptions, as compared to a low level of historical environment. Hence, hypothesis H3c was also verified.

## Conclusion and discussion

### Discussion

In the present era of internet, communication based eWOM has attracted extensive attention from the industry and academia (Zhang, 2020). It has been considered imperative for tourism enterprises to explore the social strategy of tourism public opinion management. Combining the tourists’ individual characteristics and environmental conditions can help to enhance people’s well-being experience and their personal cultural rights (Luqman et al., 2018; Zhang, 2020). Therefore, this study analyzes the tourists’ performance when they were exposed to eWOM contents. Moreover, it considered the moderating effects of individual characteristics and environmental elements to present the overall study findings. First, the interaction between eWOM perception and gender highlighted that women’s hedonic well-being score is higher, and men’s eudaimonic well-being score is higher under high eWOM perception. This suggests that in the travel selection process through eWOM content, women are more likely to seek pleasurable experiences to “top up” their happiness. Whereas men focus more on the cultural knowledge and heritage of the destination, which results in a more rational sense of self-actualization (Study 1a). Second, the interaction between eWOM perception and age, hedonic well-being was highest for older tourists, followed by young tourists, and lowest for middle-aged tourists under high eWOM perception. While eudaimonic well-being was highest for young tourists, followed by middle-aged tourists, and lowest for older tourists. It indicates that the travel selection process through eWOM content is dominated by relaxation among older tourists. Whereas young and middle-aged tourists are more interested in exploring the unknown and pursuing a sense of achievement (Study 1b). Finally, high eWOM perceptions positively affect tourists’ livelihood well-being experiences (hedonic well-being and eudaimonic well-being) in a high-level physical, social and historical environment, moderated by environmental elements. It shows that if the local scenery, customs and historical culture can be combined in the process of eWOM communication, then the happiness of tourists can be improved (Study 2).

### Theoretical implications

From a theoretical perspective, the findings of this study are important in the following ways. First, we verified the existence and applicability of livelihood well-being experiences in eWOM communication. That confirmed the tourists’ eWOM perceptions can improve well-being by conveying the emotions and connotations of the destination (Tseng et al., 2015). Tourists are enabled to find their own values and positioning (Chen et al., 2018) and satisfying their tourism demands (Le et al., 2020). Secondly, under the topic of livelihood well-being experience, this study combined the viewpoint of Vada et al. (2019) to classify livelihood well-being experience as hedonic well-being and eudaimonic well-being. Where we analyzed the effect of eWOM perception on both of the dimensions of livelihood well-being experience. Moreover, based personal characteristics (gender and age) of tourists, the study showed the impact of the interaction between eWOM perception and individual characteristics on people’s well-being experience (hedonic well-being and eudaimonic well-being). Such findings of this study helped to understand the relationship between eWOM perception and tourists’ well-being experience. The study results provided a new perspective and enriches the applicable boundaries of word-of-mouth in the tourism field. Finally, this study synthesized prior literature to expand environmental elements in terms of physical environment (Dwyer et al., 2019), social environment (Tosun et al., 2021), and historical environment (Rahmafitria et al., 2020). The experimental study conducted in combination with attractions, meet these three types of environmental conditions. It revealed the relationship between perception of eWOM and livelihood well-being experience at different environmental levels, enriching the prior research results to some extent.

### Practical implications

From a practical point of view, the findings of this study are important in the following ways. First, the study points out that eWOM perception can significantly and positively influence the livelihood well-being experience. Therefore, the tourism industry should establish online tourism websites, tourism applications, public numbers and WeChat Mini Program. This will help to gradually transfer traditional tourism services to various online tourism platforms, providing rich channels for tourists to share their knowledge, emotions, and travel experiences. In addition, the tourism service system should be continuously developed and improved, besides, a feedback mechanism should be established. Moreover, the existing deficiencies should also be corrected and improved in time, and more positive travel experiences should be provided. Thereby, it will enhance the happiness of tourists. Second, this study found that hedonic and eudaimonic well-being differed across gender and age groups in the eWOM communication process. Therefore, travel enterprises may design more personalized social media platforms. These platforms will allow travelers to not only select word-of-mouth messages that meet their own criteria, but also allow other specific groups to skim through their posted online reviews. The word-of-mouth visibility to other groups making eWOM communication more accessible and thus more targeted to meet tourists’ travel service demands. Finally, the study concludes that the interaction between eWOM perception and environmental elements has a significant impact on livelihood well-being experiences. Therefore, the tourism industry should focus on environmental rendering and encourage local residents and tourists to describe the travel environment. The description of travel environment can be in relation to the physical landscape, social flavor, or historical culture of the destination. Moreover, the local residents and tourists can express their feelings, evaluations, and suggestions about the travel in the form of text, pictures, and videos on online platforms. This is an effort to optimize travelers’ travel strategies and enhance their livelihood well-being experiences.

### Limitations and future research directions

Due to various subjective or objective reasons, this study has certain limitations. First, in terms of individual statistical characteristics, this paper only discussed the impact of the interaction between eWOM perception, gender and age on people’s livelihood well-being experience. While, other characteristics such as education, occupation and disposable income, etc. also have impact on people’s livelihood well-being experience. Therefore, the prospective follow-up studies can further explore this mechanism from the perspective of other individual characteristic elements. Second, this study only focused on the livelihood well-being experience brought about by eWOM perception. However, traditional word-of-mouth communication can also have an impact on livelihood well-being experience. That necessitates to conduct further experiments in the future to test the mechanism of the effect of traditional word-of-mouth perception and conduct comparative studies with eWOM perception (e.g., Luqman et al., 2017). Third, this study did not address the possible mediating variables involved. While eWOM perception may have an indirect effect on tourists’ happiness through a series of mediating elements (e.g., travel motivation, destination image, etc.). Thus, future studies could expand on this aspect and further refine the findings of this study.

## Data availability statement

The original contributions presented in the study are included in the article/supplementary material, further inquiries can be directed to the corresponding author.

## Author contributions

XL and YW contributed to the empirical work, the analysis of the results, and the writing of the first draft. YJ supported the total work and advised the hypotheses development. XL, YW, and YJ discussed the results and commented on the manuscript. All authors contributed to the article and approved the submitted version.

## Funding

This project was supported by Social Science Fund Special Project in Jiangxi Province (No. 22ZXHYZ11).

## Conflict of interest

The authors declare that the research was conducted in the absence of any commercial or financial relationships that could be construed as a potential conflict of interest.

## Publisher’s note

All claims expressed in this article are solely those of the authors and do not necessarily represent those of their affiliated organizations, or those of the publisher, the editors and the reviewers. Any product that may be evaluated in this article, or claim that may be made by its manufacturer, is not guaranteed or endorsed by the publisher.
